# Prospective observational study of the effectiveness of prewarming on perioperative hypothermia in surgical patients submitted to spinal anesthesia

**DOI:** 10.1038/s41598-019-52960-6

**Published:** 2019-11-11

**Authors:** Ángel Becerra, Lucía Valencia, Carlos Ferrando, Jesús Villar, Aurelio Rodríguez-Pérez

**Affiliations:** 1 Department of Anesthesiology, Hospital Universitario de Gran Canaria Doctor Negrín, Las Palmas de Gran Canaria, Spain; 2 Department of Medical and Surgical Sciences, University of Las Palmas de Gran Canaria, Gran Canaria, Spain; 3 CIBER de Enfermedades Respiratorias, Instituto de Salud Carlos III, Madrid, Spain; 4 Department of Anesthesiology, Hospital Clínic de Barcelona, Barcelona, Spain; 5 Multidisciplinary Organ Dysfunction Evaluation Research Network, Research Unit, Hospital Universitario de Gran Canaria Doctor Negrín, Las Palmas de Gran Canaria, Spain

**Keywords:** Outcomes research, Risk factors, Comorbidities

## Abstract

Prewarming has been shown to prevent intraoperative inadvertent hypothermia. Nevertheless, data about optimal prewarming-time from published clinical trials report contradictory results. We conducted this pilot study to evaluate routine clinical practice regarding prewarming and its effect on the prevalence of perioperative hypothermia in patients undergoing transurethral resection (TUR) under spinal anesthesia. This was a prospective, observational, pilot study to examine clinical practice in a tertiary hospital regarding prewarming in 140 consecutive patients. When prewarming (pw) was performed, forced-air warming was provided in the pre-anesthesia room for 15 (pw15), 30 (pw30), or 45 (pw45) min. Tympanic temperature was recorded upon entering the pre-anesthesia room, at the time of initiating surgery, and every 15 min intra-operatively. We also recorded duration of the surgical procedure and length of stay in the Post-Anesthesia Care Unit (PACU). Pw15 was performed in 34 patients, pw30 in 29 patients, and pw45 in 21 patients. Fifty-six patients did not receive pw and 96% of them developed hypothermia at the end of the surgical procedure, compared to 73% of patients in pw15 (p = 0.002), 75% in pw30 (p = 0.006) and 90% in pw45 (p = 0.3). Length of stay in the PACU was markedly shorter in pw15 (131 ± 69 min) and pw30 (123 ± 60 min) than in the non-pw group (197 ± 105 min) (p = 0.015 and p = 0.011, respectively). This difference was not significant in pw45 (129 ± 56 min) compared to non-pw patients. In conclusion, prewarming for 15 or 30 min before TUR under spinal anesthesia prevents development of hypothermia at the end of the surgical procedure.

## Introduction

Hypothermia is a frequent complication during the perioperative period in surgical patients. Its appearance can lead to deleterious effects such as surgical site infection, myocardial ischemia or bleeding^1–3^. Most studies on perioperative hypothermia have focused on patients under general anesthesia. Few studies have examined the occurrence of hypothermia in patients under spinal anesthesia despite a body temperature drop during spinal anesthesia due to the loss of temperature autoregulation, to the vasodilation secondary to sympathetic block^4–8^, and to the decrease in the shivering response^6^. Therefore, hypothermia can develop during neuraxial block, as frequently and deeply as during general anesthesia^6^.

When the negative effects of spinal anesthesia on body temperature are aggravated by other factors occurring during surgery, such as by glycine infusion during transurethral resection (TUR), temperature can decrease more profoundly. Bladder irrigation with liquids at ambient temperature can cause a decrease in body temperature of one or two degrees centigrade^9^. Prewarming (pw) associated to intraoperative warming could be beneficial in this type of patients under spinal anesthesia. In fact, the most recent clinical practice guidelines advocate for active pw before induction of general anesthesia^10–14^ (level A recommendation)^11^. However, pw in patients under spinal anesthesia is still a weak recommendation^12–14^. Most studies concerned with perioperative hypothermia in patients submitted to spinal anesthesia are focused on the importance of intraoperative active warming. Besides, those studies highlighting the importance of active prewarming do not compare different prewarming time-periods^15^. Findings of published clinical trials on this field are contradictory.

The aim of this study was to evaluate routine clinical practice and the effect of different time-periods of preoperative forced-air warming (15, 30 or 45 min) on perioperative temperature in patients submitted to TUR under spinal anesthesia. We also examined whether pw had an effect on the length of stay and the incidence of shivering in the Post-Anesthesia Care Unit (PACU) during the recovery period.

## Materials and Methods

This study was approved by the Ethics Committee at the Hospital Universitario de Gran Canaria Doctor Negrín, Las Palmas de Gran Canaria, Spain (#NAC120300) and registered at ClinicalTrials.gov (identifier NCT03527329). This is a non-randomized, pragmatic prospective study evaluating routine practice of prewarming in consecutive surgical male patients scheduled to undergo elective bladder or prostatic TUR under spinal anesthesia between March 2014 and April 2015. Exclusion criteria for enrolment into the study included: active infection, intake of antipyretics within 24 hours before surgery, neuropathy, thyroid disorders, peripheral vascular disease, skin lesions or history of hypersensitivity to skin contact devices. Female patients were also excluded in order to homogenize the sample regarding physiological and physical characteristics. Written informed consent was obtained from all patients. All methods were performed in accordance with the relevant guidelines and regulations, following good clinical practice. This manuscript adheres to the applicable STROBE guidelines.

### Temperature monitoring

For the purpose of this study, we defined hypothermia as having a body temperature lower than 36 °C^10–14^. For measuring the temperature during the perioperative phase, we used a tympanic thermometer (Genius 2 Tympanic Thermometer and Base, Covidien Ltd, Mansfield, USA). This thermometer has an accuracy of ±0.1 °C^16^. Before starting the study, nurses in charge of temperature monitoring were trained to take correct measurements. In each patient, an otoscope was used to ensure that tympanic membrane could be visualized before measurements. After checking that the probe tip was clean, a cover was placed. The probe tip was then inserted into the ear canal without an ear tug and seated in the ear canal by rotating the handle a quarter turn toward the jaw^16^. To reduce the intra-observer variability in temperature measurements, we selected the mean value of three consecutive measurements in each ear. The average perioperative body temperature was defined as the mean temperature measured from the time the patient entered into the operating room until the patient was transferred to the PACU.

### Study protocol

We collected and recorded patient’s age, body weight and height, American Society of Anesthesiologist (ASA) physical status, type of TUR (prostatic or bladder), and baseline temperature upon admittance to the hospital. On arrival at the pre-anesthesia room, core temperature was measured at the tympanic membrane (Pre-T). After this first temperature measurement, patients were prewarmed using a forced-air blanket (WarmTouch lower body blanket, Covidien Ltd, Mansfield, USA) positioned over the body and connected to a forced-air warmer (WarmTouch Model WT-5900, Covidien Ltd, Mansfield, USA). Temperature output of the warmer was set at the maximum level (43 °C).

Prewarming was applied following routine clinical practice and time was not fixed to avoid a delay in induction. The prewarming time depended on the time the patient had to wait before entering the operating room. If the duration of stay in the pre-anesthetic room was less than 5 min, the patient was included in the non-prewarmed group. The rest of patients were classified in three groups: (i) pw15: patients on pw time ≤15 min; (ii) pw30: patients on pw time ≥15 and ≤30; (iii) pw45: patients on pw time ≥30 and ≤45. Attendant anesthesiologists responsible for clinical management of patients were blinded to group assignment and had no decision over duration of prewarming.

Patient’s tympanic temperature was measured before transferring to the operating room (T0). Patients were premedicated with 1–3 mg of intravenous midazolam, at the discretion of the anesthesiologist. Once the patient entered the operating room, spinal anesthesia was performed in the sitting position using 10 mg of 0.5% hyperbaric bupivacaine intrathecally through spinous interspace L3/4 or L4/5, to reach a level of sensitive block at dermatome T10. During the surgical procedure, all patients were actively warmed using blankets over the upper part of the body. Tympanic temperature was measured at 15-min intervals from arrival into the operating room to the end of surgery (End-T). Operating room temperature during all surgical procedures was centrally controlled to be kept between 21.7–23.8 °C. Neither intravascular fluids nor bladder irrigation fluids were warmed following routine clinical practice. Room temperature, volume of intravenous fluids and volume of glycine infused were also recorded. During the surgical procedure, non-invasive arterial pressure, electrocardiography and peripheral arterial oxygen saturation were monitored in all patients.

After surgery, patients were transferred to the PACU, where the occurrence of shivering and the length of stay were recorded. Patients were treated by an independent clinician and transferred to a hospital ward once they recovered from spinal block, maintained adequate oxygen saturation, were hemodynamically stable and normothermic.

### Statistical analysis

Based on historical data, the sample size for this study was calculated using power analysis to detect a difference higher than 0.3 °C (±0.05 °C) in core temperature at the end of surgery. Nineteen patients in each group were estimated to provide 80% power for detecting a statistically significant difference at an alpha-level of 0.05. Data were analyzed using the statistics program R Core Team (R Foundation for Statistical Computing, Vienna, Austria). Results of qualitative variables are expressed as frequency and percentage. Quantitative variables are expressed as mean (SD). The Shapiro-Wilk test was used to analyze the normality of the data. To compare quantitative variables between two groups, the Student-*t* test was used in cases of variables with normal distribution, and the Mann Whitney U test when the distribution of the variables could not be adjusted to normality. To compare quantitative variables among groups, ANOVA test was used in cases of normal variables and Kruskal-Wallis in cases where distribution was not adjusted to normality. Multiple lineal regression was used for paired data to detect differences in temperature when other variables were analyzed. A p-value <0.05 was considered statistically significant.

### Study registration

Registered at ClinicalTrials.gov (Identifier: NCT03527329).

### Implication statement

Prewarming in surgical interventions is not well-standardized. We evaluated routine clinical practice using different intervals of prewarming and their effect on temperature through the perioperative period. The present study helps clarify the importance of short time-periods of prewarming on the prevention of perioperative hypothermia in patients submitted to spinal anesthesia.

## Results

A total of 140 patients were included in the study: 34 in the pw15 group, 29 in the pw30 group, 21 in the pw45 group and 56 in the non-pw group. Patient characteristics, temperature of the operating room, intravenous volume infused, duration of surgery, and amount of glycine instilled were similar in all groups (Table 1).

**Table 1:** Patient characteristics and perioperative variables.

Variable		Non-prewarmed group (n = 56)	pw15 group (n = 34)	pw30 group (n = 29)	pw45 group (n = 21)	*p*
Age (years)		69.8 (11.8)	68.7 (10.6)	72.8 (8.7)	72.4 (8.1)	0.34
Weight (kg)		79.9 (11.2)	77.8 (12.7)	80.5 (12.9)	74.8 (15.3)	0.34
BMI (kg·m^−2^)		27.6 (3.4)	26.8 (4.2)	28.3 (4.4)	26.2 (3.7)	0.21
ASA (%)	I	5.4	2.9	3.5	4.8	1
	II	35.7	29.4	34.5	38.1	
	III	48.2	58.8	55.1	47.6	
	IV	10.7	8.8	6.9	9.5	
Core temperature at hospital admission (%)	Unknown	37.5	47.0	55.2	61.9	—
	<36 °C	21.4	11.8	13.8	9.5	0.85
	>36 °C	41.1	41.2	31.0	28.6	
Operating room temperature (°C)		22.7 (0.5)	22.7 (0.4)	22.7 (0.5)	22.7 (0.3)	0.91
Duration of surgery (min)		32.9 (21.3)	32.4 (19.7)	36.2 (18.2)	40.7 (14.8)	0.37
Volume of intravenous fluids (ml)		571 (250)	589 (285)	563 (296)	657 (211)	0.59
Volume of Glycine infused (l)		9.6 (7.7)	9.9 (7.4)	9.4 (7.3)	11.8 (6.3)	0.66

Data are expressed as mean (SD) or percentage. BMI: Body Mass Index. ASA: American Society of Anesthesiologists. pw15: prewarmed for 15 min. pw30: prewarmed for 30 min. pw45: prewarmed for 45 min.

Average body temperature throughout the intraoperative period in non-pw patients was 35.35 ± 0.05 °C. Intraoperative temperature in pw15 and pw30 groups were 0.24 ± 0.08 °C and 0.36 ± 0.09 °C higher than in the non-pw group, being this difference statistically significant (p = 0.005 and p = 0.0001, respectively). Intraoperative temperature in pw45 was 0.06 ± 0.1 °C higher than in the non-pw group, but this difference did not reach statistical significance (p = 0.57). No significant relationships were found when performing the univariate analysis between average perioperative temperature and different variables, such as age (p = 0.56), BMI (p = 0.15), volume of glycine infused (p = 0.36), operating room temperature (p = 0.35) and duration of surgery (p = 0.52).

**Figure 1:**
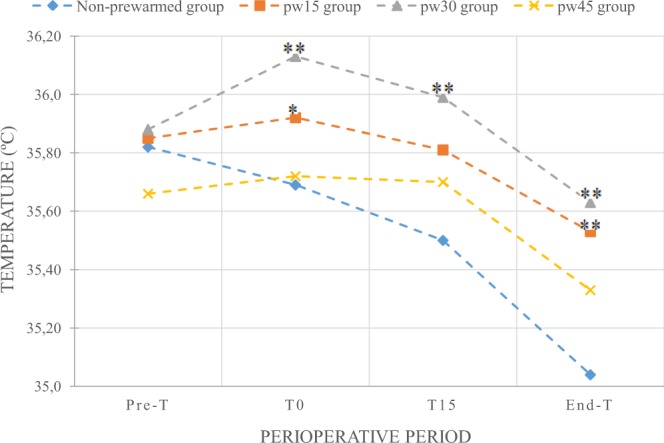
Mean perioperative temperatures (°C) in each group. pw15: prewarmed for 15 min; pw30: prewarmed for 30 min; pw45: prewarmed for 45 min; Pre-T: temperature on the arrival at the pre-anesthesia room; T0: temperature before entering the operating theatre; End-T: temperature at the end of surgery. *P = 0.02 versus non-prewarmed group. **P < 0.001 versus non-prewarmed group.

The evolution of temperature in each group is shown in Table 2 and Fig. 1. Baseline temperature of patients in the pre-anesthesia room (Pre-T) was similar in all groups. Mean body temperature of the non-pw group before entering into the operating room (T0) was 35.69 °C. After pw, T0 in pw15 and pw30 groups were 0.23 °C and 0.44 °C higher than in the non-pw group (p = 0.02 and p < 0.001, respectively). T0 in pw45 group was 0.03 °C higher than in the non-pw group, but this difference did not reach statistical significance (p = 0.83). The temperature at the end of the procedure (End-T) of the non-pw group was 35.04 °C. End-T in pw15 and pw30 were 0.49 °C and 0.59 °C higher (p < 0.001 and p < 0.001, respectively). End-T in pw45 was 0.29 °C higher than in the non-pw group, but it did not reach statistical difference (p = 0.055).

**Table 2:** Percentage of hypothermia in the pre-anesthesia room and at the end of surgery.

Temperature Measurement		Non-prewarmed (n = 56)	pw15 (n = 34)	pw30 (n = 29)	pw45 (n = 21)
Pre-T	<36 °C	55.4%	61.8%	65.5%	76.2%
	≥36 °C	44.6%	38.2%	34.5%	23.8%
End-T	<36 °C	96.4%	73.5%*	75.9%**	90.5%
	≥36 °C	3.6%	26.5%	24.1%	9.5%

pw15: prewarmed for 15 min; pw30: prewarmed for 30 min; pw45: prewarmed for 45 min; Pre-T: temperature on the arrival at the pre-anesthesia room; End-T: temperature at the end of surgery. Data are expressed as mean (SD) or percentage. *P = 0.002 versus non-prewarmed group. **P = 0.006 versus non-prewarmed group.

Most patients (96.4%) from the non-pw patients were hypothermic at the end of the procedure. This percentage decreased to 73.5% in the pw15 group (p = 0.002) and to 75.9% in the pw30 group (p = 0.006). However, hypothermia developed in 90.5% of patients in the pw45 group, without statistically significant difference from the non-pw group.

Upon admission to PACU, 42.9% of non-pw patients suffered shivering episodes. No shivering was observed in the pw15 and pw30 groups and it only affected 9.5% of patients in the pw45 group. These differences were significant in the three pw groups when compared to the non-pw group. Length of stay in PACU was shorter in the pw15 and pw30 groups when compared to the non-pw group (p = 0,015 and p = 0,011, respectively) (Table 3).

**Table 3:** Presence of shivering and length of stay in PACU in each group.

		Non-prewarmed (n = 56)	pw15 (n = 34)	pw30 (n = 29)	pw45 (n = 21)
Shivering (%)	No	57.1	100*	100*	90.5**
	Yes	42.9	0	0	9.5
Stay in PACU (min)		197 (105)	131 (69)^**+**^	123 (60)^**++**^	129 (56)

pw15: prewarmed for 15 min; pw30: prewarmed for 30 min; pw45: prewarmed for 45 min; PACU (Post-Anesthetic Care Unit). Data are expressed as mean (SD) or percentage. *P < 0.001 versus non-prewarmed group. **P = 0.006 versus non-prewarmed group. ^+^*P* = 0.015 versus non-prewarmed group. ^++^*P* = 0.011 versus non-prewarmed group.

## Discussion

To the best of our knowledge, this study is the first to report that 15 and 30 minutes of active pre-warming before TUR under spinal anesthesia decreases the prevalence of perioperative hypothermia. Our results also showed that this prewarming prevents postoperative shivering and reduces the length of stay in the PACU. Nevertheless, these benefits were not observed when prewarming lasted for 45 min.

Of note, we observed that 62.1% of patients who underwent TUR were already hypothermic upon arrival in the pre-anesthesia room, a prevalence that is higher than initially expected based on previous reports^17^. A possible explanation for a higher prevalence of preoperative hypothermia is that TUR patients are usually elderly. Moreover, males have a higher risk of developing hypothermia than women due to their physical characteristics and rate of metabolic heat production^18^. Preoperative hypothermia is a predictor for a more severe decrease in body temperature intraoperatively^12,19^. If the temperature is unknown before surgery, active measures for reversing hypothermia could be delayed. Once the temperature has decreased, its treatment is difficult since the application of heat to the body surface takes a long time to reach the core thermal compartment^20^. Intraoperative warming alone cannot avoid postoperative hypothermia^21^, and the concept of prewarming in the surgical population has been established for a long time. Active prewarming prevents hypothermia by lowering the temperature gradient between core and peripheral compartments and by reducing thermal redistribution^15,22,23^.

In our study, 15 or 30 min were able to markedly reduce the incidence of perioperative hypothermia. Our results are in agreement with other studies reporting the efficacy of prewarming in surgical procedures under neuraxial anesthesia. In elective caesarean section under epidural anesthesia, 15 min of forced-air prewarming accompanied by intraoperative active warming prevented hypothermia and shivering^24^. The same results were found applying 15 min of forced-air prewarming plus warming of intravenous fluids^25^. In contrast, a recent study showed that 20 min of prewarming plus warming of intravenous fluids was not effective at preventing hypothermia during caesarean delivery using intrathecal morphine^26^. Also, a study in a population similar to ours (men undergoing TUR under spinal anesthesia), reported that prewarming for 20 min did not reduce the incidence of hypothermia at the end of the procedure, although it reduced its severity^27^. These different results, when compared to ours, could be explained because patients with preoperative hypothermia (temperature <36 °C) were excluded in those studies. In our study, we showed that although more than 50% of patients were hypothermic at the time of arriving into the pre-anesthesia room, prewarming was effective in decreasing the incidence of hypothermia by more than 20%.

Paradoxically, we observed that the increase in body temperature was not proportional to the amount of time the prewarming was performed, since patients prewarmed during 45 min suffered more hypothermia at the end of the surgery. Our findings are consistent with other study, which show that an increased duration of prewarming beyond the 30 min in patients submitted to general anesthesia may not result in better preservation of normothermia^28^. This finding might be explained by the fact that the long time while the patient is in contact with the hot-air device could lead to excessive vasodilation, facilitating the conductance of heat to the environment^20^. Nevertheless, other factors could have influenced on having a lower temperature at the end of surgery in the pw45 group in our study. Although there were not statistically significant differences, pw45 group were submitted to a longer surgery, receiving a higher amount of Glycine and more intravenous fluid.

Prewarming for less than 30 min also decreased the length of stay in the PACU, suggesting that prewarmed patients required less time to recover their baseline temperature and could be transferred earlier to the hospital ward. The optimization of postoperative time speeds up the process of patient turnover and improves quality of care. It is important to highlight that postoperative shivering increases oxygen consumption, postoperative pain, and is one of the main causes of postoperative discomfort^4,29^.

The ideal prewarming time has long been investigated. Early studies established that a prewarming of at least 60 min was needed to prevent intraoperative hypothermia^30–34^. It was also observed that, although prewarming during 60 min did not completely prevent hypothermia, it attenuated the temperature drop^35^. Due to the inefficiency of long-time prewarming in short-term surgical procedures, studies were conducted to find out the optimal prewarming time. It was observed that in patients undergoing general anesthesia, hypothermia was reduced by using active forced-air warming for only 30 min prior to induction of anaesthesia^28,36,37^, and that even 10 min of prewarming could be effective in reducing the temperature drop and postoperative shivering^38^. These results have been confirmed in patients receiving combined general and regional anesthesia^8^.

In contrast to other studies, we did not find a relationship between hypothermia and any relevant variable, such as duration of surgery or the operating-room temperature^4,12^. The short duration of the surgery and the exposure to low room temperature during a short period of time could explain the lack of association.

Despite the strengths of our study, we acknowledge some potential limitations. First, our study was not designed as a randomized controlled trial. Dividing participants into groups depending on the time they were going to wait in the pre-anesthetic room before entering the operating room, could be the main limitation in this study, since it does not allow to ensure that the characteristics of different groups are comparable. The prewarming-time group was selected arbitrarily. However, no significant differences were found among different groups in the subsequent analysis regarding the main characteristics that may affect the thermal evolution during the perioperative period. However, we think that the main strength of this pilot study was to be able to examine different approaches regarding temperature control according to individual routine clinical practice in a Department of Anesthesia in a University Tertiary Hospital. Besides, the fact that the anesthesiologist responsible for clinical management in each participating patient did not control the prewarming time, findings are not influenced by the decision of the attending clinician. In addition, the sample size is large enough for the results to be generalizable. As it was a prospective observational study, temperature of some patients at admission was unknown and they could be already hypothermic at the beginning of the study. Because of this, clinical relevance of this study is also increased. Having only male patients as an inclusion criterion makes the sample more homogeneous in terms of physical characteristics of patients. However, the results obtained cannot be extrapolated to the general or the female population. Second, we did not measure the sensory level of the neuraxial block and, as a result, the level of sympathetic block. Considering that one of the main predictors of hypothermia during spinal anesthesia is the level of the block^5^, this variable could have conditioned the severity of hypothermia. Although all patients received intrathecally the same dose of local anesthetic, there is a possibility that other factors, such as patient’s age, spinal curvature, intra-abdominal pressure or height, could influence the level reached during spinal anesthesia. Third, the use of the non-invasive, easy accessible, and comfortable tympanic thermometer to monitor temperature could show an incidence of hypothermia different from the real measured with the gold standard of core temperature monitoring, which is through a pulmonary artery catheter. However, and following the method used in many previous studies^5,7,8,17,24–27,33,35–38^, we did not consider alternative techniques as this was a pragmatic study and temperature was measured in awake conditions.

## Conclusions

Short prewarming before TUR under spinal anesthesia reduces hypothermia appearance at the end of the surgical procedure and decreases PACU length of stay and the incidence of postoperative shivering. We encourage the implementation of actions to combat hypothermia immediately upon admission and monitoring temperature in all patients during the perioperative period.
